# Identification and Characterization of Microsatellites in Expressed Sequence Tags and Their Cross Transferability in Different Plants

**DOI:** 10.1155/2014/863948

**Published:** 2014-10-19

**Authors:** Shamshad ul Haq, Rohit Jain, Meenakshi Sharma, Sumita Kachhwaha, S. L. Kothari

**Affiliations:** ^1^Department of Botany, University of Rajasthan, Jaipur 302004, India; ^2^Department of Biosciences, Manipal University, Jaipur 302007, India; ^3^Amity Institute of Biotechnology, Amity University Rajasthan, Jaipur 302006, India

## Abstract

Expressed sequence tags (EST) are potential source for the development of genic microsatellite markers, gene discovery, comparative genomics, and other genomic studies. In the present study, 7630 ESTs were examined from NCBI for SSR identification and characterization. A total of 263 SSRs were identified with an average density of one SSR/4.2 kb (3.4% frequency). Analysis revealed that trinucleotide repeats (47.52%) were most abundant followed by tetranucleotide (19.77%), dinucleotide (19.01%), pentanucleotide (9.12%), and hexanucleotide repeats (4.56%). Functional annotation was done through homology search and gene ontology, and 35 EST-SSRs were selected. Primer pairs were designed for evaluation of cross transferability and polymorphism among 11 plants belonging to five different families. Total 402 alleles were generated at 155 loci with an average of 2.6 alleles/locus and the polymorphic information content (PIC) ranged from 0.15 to 0.92 with an average of 0.75. The cross transferability ranged from 34.84% to 98.06% in different plants, with an average of 67.86%. Thus, the validation study of annotated 35 EST-SSR markers which correspond to particular metabolic activity revealed polymorphism and evolutionary nature in different families of Angiospermic plants.

## 1. Introduction

The flowering plants are extremely diverse in their morphology, growth habit, environmental adaptation, and nuclear genome content [1]. Plant genomes tend to be large and complex, varying in size from 125 million base pairs (Mbp) for* Arabidopsis thaliana* [2] to 124,852 Mbp for* Fritillaria assyriaca* [3]. Despite so much diversity, plants do exhibit conservation of both gene content and gene order [4]. This diversity in the genomes makes comparative studies involving data from smaller genomes important for accelerating the study of larger genomes. More interesting, it can relate evolutanory consequences of diverse plant taxa. Comparison could also be made about the conserved sequences and information on the regulatory elements for extending the genetic information from model to more complicated species [5, 6]. Moreover, comparative genetic analyses have shown that different plants species comprise homologous genes for very similar functions [1, 7–9].

The DNA based markers are routinely used in ecological, evolutionary, taxonomical, comparative biology, diversity, phylogenic and genetic studies [10]. Among all the markers, microsatellites are preferred in plant genetics due to their hypervariability, relative abundance, multiallelic nature, high reproducibility, codominant inheritance, high polymorphism, high transferability, chromosome-specific location, extensive genome coverage, and highly informative and wide genomic distribution [10–12]. Microsatellites or simple sequence repeats (SSRs) are sequences in which one or few bases are tandemly repeated, ranging from 1 to 6 base pair (bp) long units which are dispersed randomly and ubiquitously throughout the genomes including both prokaryotes and eukaryotes [13–15]. Microsatellites arose from ESTs called as EST-SSRs or genic SSRs that represent functional molecular markers as “putative function or particular enzymatic activity” that can be deduced by public data base through computational approaches.

With the development of functional genomics, a huge number of expressed sequence tags (ESTs) have been deposited in the public database (NCBI) [16]. An* in silico* approach, for retrieving EST sequences from NCBI, provides a potential source of EST-SSRs, and computational methods could assign putative functions of the ESTs to various metabolic pathways. SSRs in the transcribed region are expected to be more conserved, significant, and more transferable across taxonomic boundaries than anonymous SSRs [17, 18]. Thus, the development of SSR through searching the database of EST has become a fast, efficient, and low-cost option for many studies [12, 19, 20]. The assessments of EST-SSRs, in polymorphism, diversity, and transferability have been carried out in different plant species, namely, rice [21], grape [22], sugarcane [23], tomato [24], loblolly pine [25], barley [26], rye [27], cereals [28], leguminous and nonleguminous plants [29], medicinal plants [30], and the millet and nonmillet species [31–33]. In the present study, 7630 EST sequences were retrieved from NCBI for SSR identification and characterization. Functional annotations of the sequences were assigned for the development of informative EST-SSR markers and assessment of their transferability in different families.

## 2. Material and Methods

### 2.1. Plant Material and DNA Extraction and Purification

Young juvenile, disease free, immature leaves from various therapeutic plants such as* Datura metel, Datura innoxia, Withania coagulans, Withania somnifera, Capsicum annuum, Eclipta alba, Stevia rebaudiana, Citrullus colocynthis, Ocimum sanctum, Catharanthus roseus,* and* Moringa oleifera* were collected from the University of Rajasthan campus. These plants belong to five distinct families. DNA was extracted from leaves using CTAB method [34]. DNA sample was treated with RNAase for 1 h at 37°C and purified by phenol extraction (25 phenol : 24 chloroform : 1 isoamyl alcohol, v/v/v) followed by ethanol precipitation [35] and stored at −80°C for long period. DNA was checked on a 0.8% agarose gel for confirmation of quality and concentration and final adjustments were made in 10 mM Tris HCl buffer to obtain the working concentration of 25 ng/*μ*L.

### 2.2. Mining of EST Sequences, ESTs Assembling, and Microsatellites Identification

Total 7630 putative or enzyme-encoding EST sequences were retrieved as FASTA format from the National Center for Biotechnology Information (NCBI) of different plants sources because our selected plants do not have much sequencing data in public database. ESTs assembling were carried out using CAP3 programme through online web tool (http://mobyle.pasteur.fr/cgi-bin/portal.py#forms::cap3), for identification of nonredundancy. Microsatellite identification was carried out using MISA (http://pgrc.ipk-gatersleben.de/misa/) software tool and criteria for SSRs detection were 6, 4, 3, 3, and 3 repeat units for di-, tri-, tetra-, penta-, and hexanucleotides, respectively. SSR primer pairs (forward and reverse) were designed for the selected sequence using online web tool batch primer 3 from the flanking sequences of the identified microsatellite motifs [36].

### 2.3. EST-SSR Sequences Annotation

To decipher informative assessment of SSR containing ESTs was done using Blastn/Blastx analysis for homology search and the nonredundant protein (NR) at the NCBI and functional annotation pipeline was also run at* FastAnnotator* (http://fastannotator.cgu.edu.tw/) for gene ontology (GO) system to the different GO functional classes that were displayed as horizontal bar chart in addition to detailed chart [37].

### 2.4. PCR Amplification and Electrophoresis

PCR reaction was carried out in a total of 10 *μ*L volume containing 25 ng template DNA, 1.0 *μ*L of each forward and reverse primers (at a concentration of 10 pmole/*μ*L) [31–33], 0.2 *μ*L of 100 mM of dNTPs, 0.5 U of taq DNA polymerase, 1.0 *μ*L of 10X PCR buffer, and 2.5 mM of MgCl_2_. Amplification was performed in a thermal cycler (Bio Rad, UK) in the following conditions: initial denaturation at 94°C for 5 min followed by 30 amplification cycles for denaturation for 1 min at 94°C followed by annealing for 1 min then extension for 2 min at 72°C; final extension at 72°C for 7 min was allowed. The PCR conditions particularly the annealing temperatures (varying from 52°C to 58°C) for each primer were standardized (Table 1). All the designed primers were surveyed in the selected plants, for 2-3 times, and amplified products were stored at 4°C. PCR products were used for electrophoresis on 1.5% high resolution agarose gel (Merk bioscience) at 70 V for approximately 3.5 hours, made in 0.5X TBE (Tris-Borate-EDTA) buffer. Ethidium bromide was used in agarose gel electrophoresis as intercalating dye then gel was subjected to photograph under UV light.

### 2.5. Genetic Relationship with EST-SSR Primer

Amplified bands were scored as binary data in the form of present (1) or absent (0). Dendrogram was constructed by neighbor-joining and Jaccard's algorithm using free tree/tree view free software [38, 39]. The polymorphism information content (PIC) values were calculated for each primer by using the online resource of PIC calculator (http://www.liv.ac.uk/~kempsj/pic.html).

## 3. Results

### 3.1. Frequency of Microsatellites in Expressed Sequence Tags

**Figure 1:**
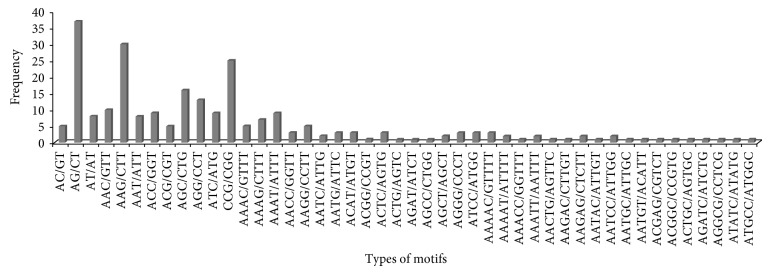
Details of motifs comprising di-, tri-, tetra- and pentanucleotides with sequence complementarity.

A total of 7630 EST sequences of putative function (enzyme-encoding sequences) involved in different plant metabolic pathways were retrieved from NCBI for microsatellite (SSR) identification. Nonredundant 1749 (1117 kb) sequences were identified comprising 884 contigs and 865 singlets, in which 263 SSRs were having 220 perfect SSRs, 38 sequences containing more than 1 SSR, and 26 SSRs present in compound formation. The frequency of EST-SSR was 3.4% or density was one SSR per 4.2 kb. Among all SSRs, trinucleotide repeats were highly abundant (47.52%) followed by tetranucleotide (19.77%), dinucleotide (19.01%), pentanucleotide (9.12%), and hexanucleotide (4.56%) repeats. A total of 58 different types of motifs were identified which belonged to three different types of dinucleotides repeats, nine different types of trinucleotides, sixteen different types of tetranucleotides, eighteen different types of pentanucleotides, and twelve different types of hexanucleotide repeats. The most frequent repeat motifs were AG/CT and AT/AT in dinucleotide, motifs AAG/CTT, CCG/CGG, and AGC/CTG in trinucleotide, motifs AAAT/ATTT and AAAG/CTTT in tetranucleotide, and motif AAAAC/GTTTT in pentanucleotide (Figure 1).

### 3.2. Expressed Sequence Tags (ESTs) Annotation and Primer Designing

**Figure 2:**
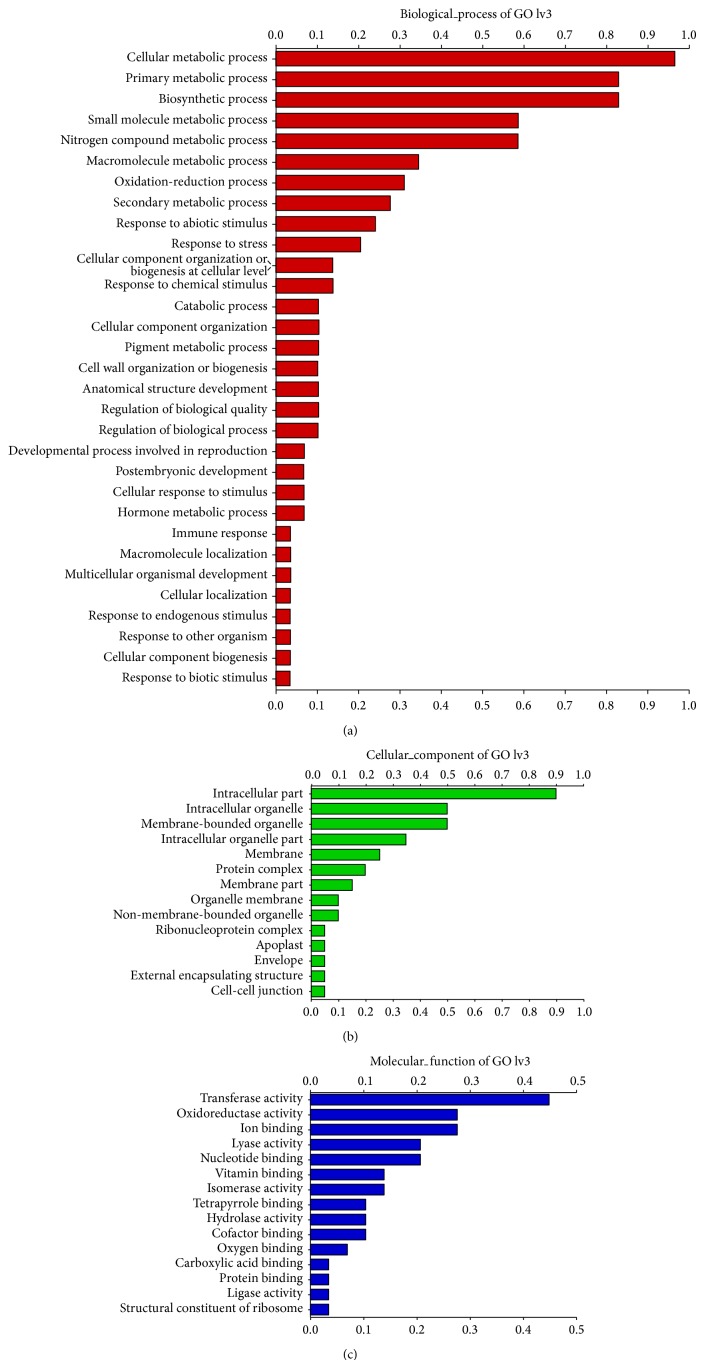
Partial results of GO annotations obtained using* FastAnnotator*. These horizontal bar charts represent the distribution of GO terms categorized as biological process (a), cellular components (b), and molecular functions (c).

EST sequences, from which the SSR markers developed, were examined by functional annotation (blastn/blastx/gene ontology) and to identify 35 EST-SSR markers, on the basis of their presence in primary metabolic process, secondary metabolic process, biosynthetic process, nitrogen compound metabolic process, oxidation-reduction process, transferase activity, oxidoreductase activity, lyase activity, nucleotide binding activity, and others (Figure 2). Primer pairs could be designed for functionally annotated 35 EST-SSRs that were 13.30% of the total microsatellites (263) identified and evaluated for polymorphic nature, cross transferability, and genetic relationships in 11 plant species of five different families. Trinucleotide repeats were highly abundant in 35 EST-SSRs followed by tetra- and dinucleotide repeats (Table 1). All these were associated with common metabolic pathways such as GO:0009813 flavonoid biosynthetic process, GO:0045430 chalcone isomerase activity, GO:0016114 terpenoid biosynthetic process, GO:0004452 isopentenyl-diphosphate delta isomerase activity, GO:0046653 tetrahydrofolate metabolic process, GO:0004489 methylenetetrahydrofolate reductase (NADPH) activity, GO:0006694 steroid biosynthetic process, GO:0008483 transaminase activity, GO:0000162 tryptophan biosynthetic process, GO:0006571 tyrosine biosynthetic process, GO:0009094 L-phenylalanine biosynthetic process, GO:0006633 fatty acid biosynthetic process, GO:0009809 lignin biosynthetic process, GO:0009695 jasmonic acid biosynthetic process, GO:0004310 farnesyl-diphosphate farnesyltransferase activity, GO:0004311 farnesyltranstransferase activity, GO:0004713 protein tyrosine kinase activity, GO:0045548 phenylalanine ammonia-lyase activity, GO:0009821 alkaloid biosynthetic process, and GO:0006695 cholesterol biosynthetic process (see supplementary table available online at http://dx.doi.org/10.1155/2014/863948).

### 3.3. Amplification and Polymorphism of Annotated EST-SSR Markers in Selected Plants

**Figure 3:**
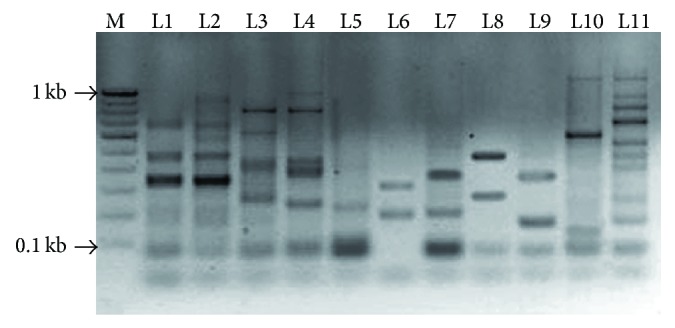
PCR amplification of ESM-28 primer in eleven plants belonging to five different families. Lane 1* Datura metel*, 2* Datura innoxia*, 3* Withania coagulans*, 4* Withania somnifera*, 5* Capsicum annuum*, 6* Stevia rebaudiana*, 7* Eclipta alba*, 8* Citrullus colocynthis*, 9* Ocimum sanctum*, 10* Catharanthus roseus*, and 11* Moringa oleifera*.

A set of 35 primer pairs from different microsatellites in EST was tested for PCR optimization, characterization, and amplification with 11 plants belonging to different families. All markers produced polymorphic amplification profile in selected plants (Figure 3), which ranged from 50 to 1050 bp. DNA finger printing data of 35 EST-SSR with eleven plants revealed a total of 402 alleles at 155 loci with an average of 2.6 alleles per locus. The markers designed in this study had potential of showing polymorphism among different plants and the polymorphic information content (PIC) of 35 EST-SSR ranged from 0.15 to 0.93 with an average 0.77.

### 3.4. Cross Transferability

All 35 annotated EST-SSR markers were assessed for cross transferability in the selected plants. The cross transferability of these markers was found to be 86.45% in* Datura metel*, 81.29% in* Datura innoxia*, 96.77% in* Withania coagulans*, 98.06% in* Withania somnifera*, 85.16% in* Capsicum annuum*, 34.84% in* Stevia rebaudiana*, 49.68% in* Eclipta alba*, 54.19% in* Citrullus colocynthis*, 43.23% in* Ocimum sanctum*, 58.71% in* Catharanthus roseus*, and 58.66% in* Moringa oleifera*, with an average of 67.86% (Table 2). These markers were found to be more transferable in Solanaceous plants (*Datura metel, Datura innoxia, Withania coagulans, Withania somnifera, Capsicum annuum*), ranging from 81.29% to 98.06% with an average of 89.55% as compared to other plants showing variable transfer rates. Thus, all markers showed reliable amplification pattern in different plants and were scored as transferable.

### 3.5. Genetic Diversity Analysis by EST-SSRs

**Figure 4:**
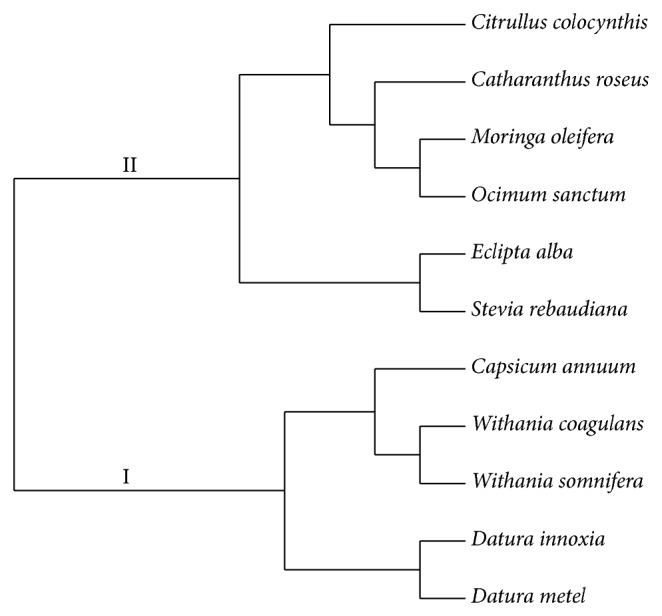
A dendrogram of genetic relationships revealed by 35 annotated EST-SSR markers, based on neighbor-joining and Jaccard's algorithm using free tree and tree view software.

Genetic relationship among selected plants was further analyzed by construction of dendrogram through allelic data obtained from EST-SSR primer amplification. All the plants were grouped into two major clusters. Cluster I contained 5 plants of Solanaceae family with two subgroups (Ia and Ib); each subgroup comprised same genus plants clustered together (*Datura metel*,* Datura innoxia* (Ia) and* Withania coagulans*,* Withania somnifera* (Ib)). Cluster II contained 6 plant species classified into two major subgroups (IIa and IIb). Subgroup IIa comprised Asteraceous plants (*Eclipta alba* and* Stevia rebaudiana*) clustered together and subgroup IIb comprised four plant species into three separate edges of the dendrogram, exception with one plant (Figure 4). Thus, the annotated 35 EST-SSR markers showed discriminatory potential to some extent and showed close intimacy amongst Solanaceous and between Asteraceous plants.

## 4. Discussion

The present study intended to utilize publicly available EST sequences from different plant sources for functional annotation of EST sequences to decode informative EST-SSR markers using* in silico* approach. Experimental methods to develop SSR markers are laborious, time consuming, and expensive; therefore use of publicly available EST libraries which reduce time and expenses is now being used as an alternative for marker identification [16, 20, 40, 41]. We identified nonredundant 263 microsatellites having di-, tri-, tetra-, penta-, and hexanucleotide repeats. The SSR frequency in the ESTs collection was 3.4% which is close to earlier reports in other plants species, namely, 3.4% in* Physcomitrella patens* and 3.5% in* Oryza sativa* [20] and 3.2% in cereals [42] and 4.1% in almond [43]. Other studies also reported SSRs in various frequencies, namely, 2.5% in grapes [22], 2.88% in sugarcane [23], 4.7% in rice [44], and 2.8% in barely [45]. In general, about 5% of ESTs contained SSRs in diverse plant species [46]. The differences in the frequency of EST-SSRs could be attributed to the “search criteria” used, type of SSR motif, size of sequence data, and the mining tools used [31, 47]. An average density of one SSR per 4.2 kb was detected which is closely comparable to earlier reported in date palm [48] and in cereals [42].

**Figure 5:**
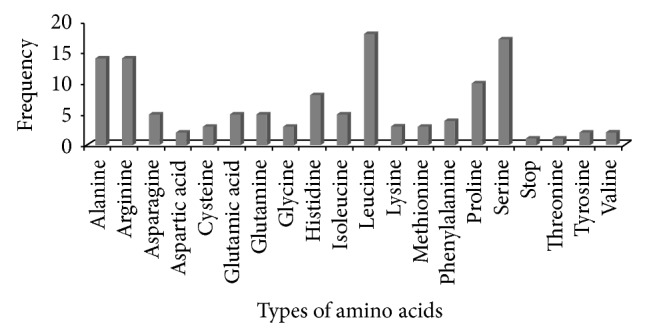
Details of predicted amino acids encoded by trinucleotide motifs.

Among 263 microsatellites, trinucleotide repeat motifs were the most abundant, with a frequency of 47.52% followed by tetra- (19.77%), di- (19.01%), penta- (9.12%), and hexanucleotide (4.56%) repeats. Varshney et al. [42] reported that trinucleotide repeats (TNRs) are the most common, followed by either dinucleotide repeats (DNRs) or tetranucleotide (TTNRs) repeats. Our result of trinucleotide repeat frequency is in close agreement with previous studies reporting 48.5% in sugarcane [49] and 48% in* Setaria italic* [32]. Some other studies also reported high TNRs, namely, cereals [50],* Ricinus communis* [51],* Eucalyptus globulus* [52], sugarcane [12], and* Setaria italica* [31]. The reason for the abundance of trinucleotide repeats in plants might be attributed to absence of frameshift mutations [53]. Among all types of trinucleotide motifs, AAG/CTT, CCG/CGG, and AGC/CTG were in high proportion. Motifs GGA/TTC, CCT/AGG, GAA/TTC, and CCG/GGC were also detected. These motifs can form hairpin-like structures, which stabilize and allow them to escape from repair mechanisms [15, 54]. Each trinucleotide motif encodes a particular amino acid including stop codon which participates within protein in various metabolic activities [20, 55]. Predictable, twenty different types of amino acids were detected in trinucleotide motifs including one stop codon (Figure 5). Amino acids (leucine, serine, alanine, and arginine) encoded by trinucleotide motifs are in agreement with earlier studies [20, 30, 55, 56].

According to functional annotation, 35 EST-SSRs were identified due to their direct involvment in metabolic pathways through blastn/blastx and gene ontology (GO). As observed in earlier studies, relavant transcripts were detected using functional annotation pipelines for various applications [57]. Most of these were involved in biological processes and molecular function such as primary metabolism, secondary metabolism, nitrogen compound metabolism, oxidation-reduction process, and transferase activity. The 35 EST-SSR primer pairs were designed and surveyed in different plants. All primers produced clear PCR amplification profiles in all the selected plants and produced 402 alleles at 155 loci with an average of 2.6 alleles/locus. This result is in close agreement with earlier study reported in chickpea (2.6 alleles/locus) [58].

A set of 35 EST-SSR markers produced a clear amplification profile and these were found to be transferable among the selected plant species. The frequency of cross transferability ranged from high in* W*.* somnifera* (98.06%) to a low (34.84%) in* S*.* rebaudiana *with an average of 67.86%. This result is in conformity with earlier report on cross transferability of* Medicago truncatula* EST-SSRs into four leguminous and 3 non-leguminous plants [29]. The transferability (70%) of castor bean SSRs was reported in* J. curcas* and other* Jatropha* species [59]. Mishra et al. [30] reported cross transferability (31–57%) of Madagascar periwinkle EST-SSR markers in other medicinal plants. Choudhary et al. [58] also observed cross transferability (68.3% to 96.6%) of chickpea EST-SSR marker across 6 annual* Cicer* species and also reported 29.4% to 61.7% transferability in seven legume genera. Foxtail millet derived EST-SSR markers showed cross transferability of approximately 85 to 89% in different types of millets and nonmillets [31–33]. Saha et al. [60] also reported approximately 92% transferability from tall fescue to 7 grass species. Some other higher level of transferability was reported in other studies, namely, 86.6% transferability of wheat EST-SSRs to other cereal plants [28], 96.5% cross species amplification among 22* Gossypium* species [61], 95.2% cross transferability between* Saccharum* complex and cereals [49], and 90% transferability of* Vigna radiata* derived EST-SSR in other* Vigna* species [62]. Some Lower frequency of transferability was also reported in earlier studies. Gutierrez et al. [63] reported that approximately 40.6% transferability of* Medicago truncatula* EST-SSR markers amplified across 3 pulse crops (faba bean, chickpea, and pea). In this study, 35 EST-SSR markers were found to be more transferable (89.54%) among Solanaceous plant species than other plant taxa and these markers can give credence to various genetic applications in Solanaceous plants.

Further, the genetic relationships among the eleven plants species were evaluated by construction of dendrogram (neighbor-joining/jaccard's algorithm) using allelic data amplified through 35 EST-SSR markers. Here these markers showed close intimacy amongst Solanaceous plants (*D. metel, D. innoxia, W. coagulans, W. somnifera, *and* C. annuum*) and between Asteraceous plants (*E. alba and S. rebaudiana*) and also showed discrimination to some extent in other selected plants (*C. colocynthis, O. sanctum, C. roseus, *and* M. oleifera*). Similar relationship was shown by Gupta and Prasad [29] who evaluated the genetic relationships between leguminous (*M. truncatula*, lentil, pea, and chickpea) and nonleguminous plants (*A. thaliana*, tomato, wheat). Some other studies also reported genetic relationships using EST-SSR markers in other plant species such as in bread wheat [50], Grasses [60], sugarcane [49] and millets and nonmillets [31–33].

## 5. Conclusion

This study revealed the insight of abundance and distribution of microsatellites in the expressed sequence tags, retrieved from public data base. Further, functional annotation was feasible to develop and select the informative EST-SSR markers for various genomic applications. This is a bypass approach to reduce cost and time and it is an efficient way to analyze the transcribed portion of genome besides development of own libraries. Finally, 35 EST-SSR markers were developed and experimentally validated for their polymorphic nature, cross transferability, and genetic relationship in eleven different plants species. On the basis of amplification profiles, all these markers were found to be transferable. Genetic relations were established to unambiguously differentiate selected plants species.

## Supplementary Material

Supplementary Table: The complete details of most promising hits of gene ontology of 35 EST-SSRs given in the supplementary table.

## Figures and Tables

**Table 1:** Details of 35 EST-SSR primer pairs sequences.

Serial number	Type	Primer pairs sequence	Ta (°C)	SSR	PIC value	*E* value	Putative identities
EMS-1	F	CTCAGAGTTTGCTCACCCCTAC	56	(GAA)_4_	0.834	1.74*E* − 11	Chalcone-flavonone isomerase
EMS-1	R	TGCTGGGAAATGGTATGTGA					
EMS-2	F	TTAGACTGTGACACTGCGAAGC	52	(CAA)_4_	0.91	9.59*E* − 81	Chalcone synthase
EMS-2	R	ACTTGGCCGAAAAACAACAC					
EMS-3	F	CCACCCACTAAATCACTTGACA	52	(CCT)_4_	0.748	2.6*E* − 113	Cinnamate 4-hydroxylase
EMS-3	R	GATCCTCCTCGCTCTCGAAT					
EMS-4	F	AGCCACTGCACTTCTTGTTCTT	52	(GCC)_7_	0.2552	6.41*E* − 38	Cinnamyl alcohol dehydrogenase
EMS-4	R	GGGATCTTCACCACAAACTTCT					
EMS-5	F	CGTGGTGGAAAAGCTATTTAGG	56	(AAT)_4_	0.7268	2.62*E* − 82	Flavonol synthase
EMS-5	R	AAACAAAACCACCCACTTCA					
EMS-6	F	AATCATGGCCCTTTACCCTAGT	52	(CTTT)_3_	0.1516	2.19*E* − 68	Endoxyloglucan transferase
EMS-6	R	CTTTGGCTTCCATTGTTCTTGT					
EMS-7	F	GAAACTGAGGCACACAAAAA	52	(TTC)_4_	0.8979	2.55*E* − 76	Endoxyloglucan transferase
EMS-7	R	TCATCAGCGTTCCATAGACTGT					
EMS-8	F	GAAGAAACGCACTCTCTCTCTCTT	58	(CTCT)_3_	0.7207	2.72*E* − 18	Oligosaccharyl transferase
EMS-8	R	ATATGAAGGGACCATAGCCAGA					
EMS-9	F	GTCGTTGCTTCTTTCTGCTTTT	53	(CTTT)_3_	0.7064	1.03*E* − 62	Xyloglucan transferase
EMS-9	R	TAAGGGTGGGTAAATGGGATAC					
EMS-10	F	CGTACGAGTTGAATCTCAGGA	56	(GAGA)_4_	0.6197	9.17*E* − 80	Glutamine synthetase
EMS-10	R	CCAACAGGCCAGTTCACTTC					
EMS-11	F	CCAACAACCACAAGAAACTGG	56	(CCAA)_3_	0.8481	7*E* − 63	Isoflavone reductase
EMS-11	R	ACGACCTCCGAACATTCAAC					
EMS-12	F	CACATGCAACAGCACATCAAT	56	(CTT)_6_	0.9244	5.63*E* − 68	Isopentenyl-diphosphate isomerase
EMS-12	R	CATCGACTGGTACATCTTCAGC					
EMS-13	F	AGGGGAGAGGAAGGTGGAGT	52	(TGC)_4_	0.8996	5.34*E* − 22	Isopentenyl-diphosphate delta isomerase
EMS-13	R	CAATTTCATGTTCTCCCCAGAT					
EMS-14	F	AATCAGAAAATGGCACAGTCCT	54	(CTCT)_3_	0.6221	6.61*E* − 28	Isopentenyl pyrophosphate
EMS-14	R	TCAGAAGGTGCCTTGTAAAGAA					
EMS-15	F	GTTAAAGGTTCAGGAACGAACG	54	(CTT)_4_	0.777	2.41*E* − 38	Isopentenyl pyrophosphate
EMS-15	R	GCAGATAGTCCAGTTCATGCTC					
EMS-16	F	GTTAAAGGTTCAGGAACGAACG	54	(CTT)_4_	0.828	8.73*E* − 36	Dimethyllallyl pyrophosphate isomerase
EMS-16	R	AACAGGTGTTTGTCCACACAAG					
EMS-17	F	GTTAAAGGTTCAGGAACGAACG	54	(CTT)_4_	0.8344	1.3*E* − 20	Dimethyllallyl pyrophosphate isomerase
EMS-17	R	CAACAATACCAAGCTCATCCAA					
EMS-18	F	CACGAGGCTTTTTGAGAAAT	54	(CTAG)_3_	0.4159	2.05*E* − 45	Isopentenyl transferase
EMS-18	R	CCCCATAAAAAGCAACAGTCAT					
EMS-19	F	CAATAATTAATCCAGGCGGTTC	52	(TGGC)_3_	0.8307	6.23*E* − 55	Enoyl-[acyl-carrier-protein]
EMS-19	R	CAAAGACATCGGTGATTCAGTG					
EMS-20	F	GATCAAGGAATGCCGATCTTAC	52	(TTC)_3_	0.8333	1.86*E* − 36	Phenylalanine ammonia-lyase
EMS-20	R	CGAGGTGCCATTTTATTTCC					
EMS-21	F	AATCCTCCTCCTCCTTCCTC	52	(CGC)_6_	0.8425	3*E* − 18	Farnesyltransferase alpha subunit
EMS-21	R	CGCTATGAGACCAAGCATGTAA					
EMS-22	F	ACGATTGTTTGATTGCCAAG	52	(GGAT)_3_	0.8892	4.94*E* − 52	Alanine aminotransferase
EMS-22	R	TCCAAATGTTCCAGCACAAA					
EMS-23	F	GCACCTTTTCAACCGTTCAC	52	(CTT)_4_	0.8966	5.32*E* − 88	Alanine aminotransferase
EMS-23	R	GGCTTGCACCATCTGTCAT					
EMS-24	F	CCTCTCTCTCCCTCTCTCCAA	54	(CAC)_4_	0.9127	1.44*E* − 07	Isopropylmalate dehydratase
EMS-24	R	AAATATCCGAGGGTACGATTCC					
EMS-25	F	CATTTTCCTTGCCTCTCTCTCT	54	(CGC)_8_	0.8831	7*E* − 29	Chorismate mutase
EMS-25	R	CACTGAGCGCATGTCTTTTG					
EMS-26	F	CGCTCTAGTTTCACATAAGCAGTC	54	(TCTT)_3_	0.7855	1.74*E* − 11	Serine/threonine-protein kinase
EMS-26	R	GAATCATTTGCTTTTGGGTGTC					
EMS-27	F	AGGAAGGAAGGAAGGAGAGGAG	56	(CCG)_4_	0.828	1.93*E* − 24	Putative thiolase
EMS-27	R	CTGAGAAAACAGCTCAACTTGG					
EMS-28	F	GCTTCCGTCTCCTTGGATAAC	52	(GTC)_4_	0.9231	3.18*E* − 15	Acetyl-CoA acyltransferase
EMS-28	R	AACAGAACAAGCCCAGAACATT					
EMS-29	F	CCAACTCCTCTCAACTTCTTGAT	53	(CGG)_4_	0.8525	1.6*E* − 101	Putative alanine transaminase
EMS-29	R	GGGAAATGCCAGCAGAATAA					
EMS-30	F	CAAAGGCGGATGAGTTCAAGT	56	(TGG)_3_	0.9252	1.2*E* − 115	Flavonoid 3′-hydroxylase
EMS-30	R	CCATACGTTGACCAAGAGAGTG					
EMS-31	F	TGTCTACTATCCAGCGAAACCA	54	(AAG)_3_	0.7549	3*E* − 18	Cytochrome p450 monooxygenase
EMS-31	R	CAGCTAGTAAGATCGAGTTCAAACA					
EMS-32	F	AAGCTATACGGCCCGATTTT	52	(AGA)_3_	0.861	1.28*E* − 21	Cytochrome p450 monooxygenase
EMS-32	R	TTTGATGGAGAAAGGTTGGTCT					
EMS-33	F	TTGAAATCTGGTCAAGAGGATG	52	(TA)_8_	0.5896	4.81*E* − 13	Methylenetetrahydrofolate reductase
EMS-33	R	AAGGCTGCACTTTGTATTGTCC					
EMS-34	F	TGGGACATGTGCTATTTGCTAC	52	(ACTG)_3_	0.8991	1.00*E* − 79	Squalene synthase
EMS-34	R	GCAGGTGAGAGCCAAGATAAAT					
EMS-35	F	ACACAACAGGATCCTTCGAAAT	54	(AT)_5_	0.8722	2.18*E* − 39	Putative tryptophan synthase alpha chain
EMS-35	R	AAGCGAGGCTGCCAAGAAC					

**Table 2:** Details of cross transferability of 35 EST-SSR markers in eleven plants belonging to five different families.

Serial number	Amplification range	*Datura metel *	*Datura innoxia *	*Withania coagulans *	*Withania somnifera *	*Capsicum annuum *	*Eclipta alba *	*Stevia rebaudiana *	*Citrullus colocynthis *	*Ocimum sanctum *	*Catharanthus roseus *	*Moringa oleifera *	Primer transferability
EMS-1	104–891	8	3	4	3	4	2	0	3	1	2	1	90.91
EMS-2	129–884	0	8	7	9	7	6	0	3	6	3	4	81.82
EMS-3	117–794	6	4	2	2	1	4	1	3	1	1	2	100.00
EMS-4	92–358	2	2	1	2	1	1	1	1	1	1	1	100.00
EMS-5	135–802	3	4	4	2	3	2	1	1	1	1	2	100.00
EMS-6	94–164	2	2	2	0	2	1	1	1	1	1	1	90.90
EMS-7	105–919	1	2	9	2	5	7	1	2	1	8	2	100.00
EMS-8	98–680	6	2	2	1	3	1	1	2	1	4	1	100.00
EMS-9	130–717	3	2	5	3	2	1	3	1	2	2	1	100.00
EMS-10	92–913	5	2	3	2	2	1	1	2	2	1	1	100.00
EMS-11	135–724	5	3	5	4	5	0	1	4	1	1	2	90.90
EMS-12	121–881	7	6	3	11	4	3	2	8	5	5	7	100.00
EMS-13	75–629	0	7	8	9	2	7	2	5	2	6	0	81.81
EMS-14	106–649	3	2	2	3	0	1	1	0	0	1	0	63.64
EMS-15	99–517	5	3	0	2	2	1	1	5	1	3	2	90.90
EMS-16	105–853	5	4	2	6	3	5	2	1	2	3	1	100.00
EMS-17	77–930	4	3	7	5	6	3	2	2	1	1	1	100.00
EMS-18	83–787	1	2	4	2	3	0	1	2	1	1	4	90.90
EMS-19	111–603	3	2	9	7	2	2	1	4	1	3	3	100.00
EMS-20	117–560	5	4	10	4	6	1	1	3	2	4	1	100.00
EMS-21	101–863	6	5	6	5	4	1	1	2	1	2	1	100.00
EMS-22	108–774	3	6	7	5	6	2	0	1	2	6	7	90.90
EMS-23	101–837	0	3	6	6	10	1	1	7	3	3	8	90.90
EMS-24	61–785	2	4	3	5	5	1	0	5	6	5	5	90.90
EMS-25	124–790	5	7	9	7	5	1	2	0	3	2	6	90.90
EMS-26	50–425	7	3	3	4	4	1	1	1	1	2	1	100.00
EMS-27	124–564	5	4	4	4	0	3	0	4	1	1	1	81.81
EMS-28	50–1000	4	4	5	6	2	2	3	2	3	4	9	100.00
EMS-29	91–504	2	2	4	6	10	4	2	1	2	1	1	100.00
EMS-30	139–900	8	7	6	7	4	4	0	2	6	6	5	90.90
EMS-31	97–388	4	1	2	0	2	2	0	1	1	2	3	81.81
EMS-32	84–774	2	4	5	7	5	1	0	2	1	0	2	81.81
EMS-33	119–419	4	1	5	2	2	1	1	1	1	1	1	100.00
EMS-34	117–795	5	5	11	5	7	1	1	2	1	2	1	100.00
EMS-35	127–951	3	3	3	4	3	3	0	0	2	2	2	81.81
		134	126	150	152	132	77	54	84	67	91	90	
Percent of transferability of each plant		**86.45**	**81.29**	**96.77**	**98.06**	**85.16**	**49.68**	**34.84**	**54.19**	**43.23**	**58.71**	**58.06**	**67.86**%
