# Supplementary material for: Identification and Characterization of Microsatellites in Expressed Sequence Tags and Their Cross Transferability in Different Plants
Source: Int J Genomics. 2014 Oct 19;2014:863948. doi: 10.1155/2014/863948 (PMC4217358; doi:10.1155/2014/863948)
Supplement: Supplementary file 1 — Supplementary Table: The complete details of most promising hits of gene ontology of 35 EST-SSRs given in the supplementary table. [file 863948.f1.pdf]

Supplementary table: Details of best promising Hits of ESTs having SSR were obtained through Fastannotator.

Serial No	Entry	Biological Process	Cellular Component	Molecular Function
1	gi|17519141|gb|BM188183.1|BM188183	GO:0009813 flavonoid biosynthetic process<br>GO:0042398 cellular modified amino acid biosynthetic process<br>GO:0080167 response to karrikin		GO:0045430 chalcone isomerase activity
2	gi|54682355|gb|CV656076.1|CV656076	GO:0009813 flavonoid biosynthetic process		GO:0016210 naringenin-chalcone synthase activity
3	gi|71820633|gb|DR998024.1|DR998024	GO:0055114 oxidation-reduction process<br>GO:0006558 L-phenylalanine metabolic process<br>GO:0009805 coumarin biosynthetic process<br>GO:0009809 lignin biosynthetic process<br>GO:0009811 stilbene biosynthetic process<br>GO:0006118 electron transport		GO:0020037 heme binding<br>GO:0016710 trans-cinnamate 4-monooxygenase activity<br>GO:0009055 electron carrier activity
4	gi|25799294|gb|CA755255.1|CA755255	GO:0009809 lignin biosynthetic process<br>GO:0055114 oxidation-reduction process<br>GO:0009805 coumarin biosynthetic process<br>GO:0009811 stilbene biosynthetic process		GO:0045551 cinnamyl-alcohol dehydrogenase activity<br>GO:0000166 nucleotide binding<br>GO:0008270 zinc ion binding<br>GO:0052747 sinapyl alcohol dehydrogenase activity
5	gi|56431124|gb|CX023192.1|CX023192	GO:0051555 flavonol biosynthetic process<br>GO:0055114 oxidation-reduction process	GO:0005737 cytoplasm	GO:0045486 naringenin 3-dioxygenase activity<br>GO:0045431 flavonol synthase activity<br>GO:0031418 L-ascorbic acid binding<br>GO:0005506 iron ion binding<br>GO:0016702 oxidoreductase activity, acting on single donors with incorporation of molecular oxygen, incorporation of two atoms of oxygen
6	gi|226808327|gb|GO563021.1|GO563021	GO:0009414 response to water deprivation<br>GO:0006073 cellular glucan metabolic process	GO:0005618 cell wall<br>GO:0048046 apoplast	GO:0016762 xyloglucan:xyloglucosyl transferase activity<br>GO:0033946 xyloglucan-specific endo-beta-1,4-glucanase activity
7	gi|209724892|gb|BW669581.1|BW669581	GO:0006073 cellular glucan metabolic process<br>GO:0007047 cellular cell wall organization	GO:0005618 cell wall<br>GO:0048046 apoplast	GO:0016762 xyloglucan:xyloglucosyl transferase activity<br>GO:0004553 hydrolase activity, hydrolyzing O-

				glycosyl compounds
8	gi|209724609|gb|BW677716.1|BW677716	GO:0009651 response to salt stress<br>GO:0018279 protein N-linked glycosylation via asparagine	GO:0005886 plasma membrane<br>GO:0008250 oligosaccharyltransferase complex	GO:0004579 dolichyl-diphosphooligosaccharide-protein glycotransferase activity
9	gi|55857142|gb|CV881934.1|CV881934	GO:0009414 response to water deprivation<br>GO:0006073 cellular glucan metabolic process	GO:0005618 cell wall<br>GO:0048046 apoplast	GO:0016762 xyloglucan:xyloglucosyl transferase activity<br>GO:0033946 xyloglucan-specific endo-beta-1,4-glucanase activity
10	gi|27387337|gb|CA900345.1|CA900345	GO:0009399 nitrogen fixation<br>GO:0006542 glutamine biosynthetic process<br>GO:0009252 peptidoglycan biosynthetic process	GO:0005737 cytoplasm	GO:0004356 glutamate-ammonia ligase activity<br>GO:0005524 ATP binding
11	gi|56162853|gb|CV986979.1|CV986979	GO:0055114 oxidation-reduction process		GO:0033788 leucoanthocyanidin reductase activity<br>GO:0000166 nucleotide binding
12	gi|254066103|gb|GR726006.1|GR726006	GO:0009240 isopentenyl diphosphate biosynthetic process<br>GO:0015995 chlorophyll biosynthetic process<br>GO:0009908 flower development<br>GO:0016114 terpenoid biosynthetic process<br>GO:0009060 aerobic respiration<br>GO:0006096 glycolysis<br>GO:0046686 response to cadmium ion<br>GO:0006694 steroid biosynthetic process	GO:0005829 cytosol<br>GO:0005739 mitochondrion<br>GO:0009507 chloroplast	GO:0004452 isopentenyl-diphosphate delta-isomerase activity<br>GO:0016787 hydrolase activity
13	gi|182974060|gb|FF599768.1|FF599768	GO:0035634 response to stilbenoid<br>GO:0006695 cholesterol biosynthetic process<br>GO:0016114 terpenoid biosynthetic process	GO:0005777 peroxisome<br>GO:0005829 cytosol<br>GO:0005739 mitochondrion	GO:0030145 manganese ion binding<br>GO:0016787 hydrolase activity<br>GO:0000287 magnesium ion binding<br>GO:0004452 isopentenyl-diphosphate delta-isomerase activity
14	gi|209712696|gb|BW684626.1|BW684626	GO:0015995 chlorophyll biosynthetic process<br>GO:0009908 flower development<br>GO:0015979 photosynthesis<br>GO:0006694 steroid biosynthetic process<br>GO:0016114 terpenoid biosynthetic process	GO:0009507 chloroplast<br>GO:0005829 cytosol<br>GO:0005739 mitochondrion	GO:0046872 metal ion binding<br>GO:0016787 hydrolase activity<br>GO:0004452 isopentenyl-diphosphate delta-isomerase activity
15	gi|209728353|gb|BW659990.1|BW659990	GO:0009240 isopentenyl diphosphate biosynthetic process<br>GO:0015995 chlorophyll biosynthetic process	GO:0005829 cytosol<br>GO:0005739 mitochondrion	GO:0004452 isopentenyl-diphosphate delta-isomerase activity<br>GO:0016787 hydrolase activity

		GO:0009908 flower development<br>GO:0050992 dimethylallyl diphosphate biosynthetic process<br>GO:0009060 aerobic respiration<br>GO:0006096 glycolysis<br>GO:0015979 photosynthesis<br>GO:0046686 response to cadmium ion<br>GO:0006694 steroid biosynthetic process<br>GO:0016114 terpenoid biosynthetic process	GO:0009536 plastid	
16	gi|209727623|gb|BW663783.1|BW663783	GO:0009240 isopentenyl diphosphate biosynthetic process<br>GO:0015995 chlorophyll biosynthetic process<br>GO:0009908 flower development<br>GO:0050992 dimethylallyl diphosphate biosynthetic process<br>GO:0016114 terpenoid biosynthetic process<br>GO:0015979 photosynthesis<br>GO:0006694 steroid biosynthetic process	GO:0005829 cytosol<br>GO:0005739 mitochondrion<br>GO:0009507 chloroplast	GO:0004452 isopentenyl-diphosphate delta-isomerase activity<br>GO:0016787 hydrolase activity
17	gi|209712023|gb|BW668424.1|BW668424	GO:0009240 isopentenyl diphosphate biosynthetic process<br>GO:0015995 chlorophyll biosynthetic process<br>GO:0009908 flower development<br>GO:0050992 dimethylallyl diphosphate biosynthetic process<br>GO:0009060 aerobic respiration<br>GO:0006096 glycolysis<br>GO:0015979 photosynthesis<br>GO:0046686 response to cadmium ion<br>GO:0006694 steroid biosynthetic process<br>GO:0016114 terpenoid biosynthetic process	GO:0005829 cytosol<br>GO:0005739 mitochondrion<br>GO:0009536 plastid	GO:0004452 isopentenyl-diphosphate delta-isomerase activity<br>GO:0016787 hydrolase activity
18	gi|71824350|gb|DT001742.1|DT001742	GO:0034265 isopentenyl adenine biosynthetic process<br>GO:0008033 tRNA processing	GO:0005634 nucleus<br>GO:0009536 plastid	GO:0052622 ATP dimethylallyltransferase activity<br>GO:0005524 ATP binding<br>GO:0009824 AMP dimethylallyltransferase activity<br>GO:0052623 ADP dimethylallyltransferase activity
19	gi|226770420|gb|GO523629.1|GO523629	GO:0006633 fatty acid biosynthetic process<br>GO:0055114 oxidation-reduction process<br>GO:0042967 acyl-carrier-protein biosynthetic process	GO:0005835 fatty acid synthase complex<br>GO:0009941 chloroplast envelope<br>GO:0009570 chloroplast stroma<br>GO:0009579 thylakoid	GO:0016631 enoyl-[acyl-carrier-protein] reductase activity<br>GO:0005507 copper ion binding<br>GO:0004318 enoyl-[acyl-carrier-protein] reductase (NADH) activity<br>GO:0000166 nucleotide binding

20	gi|5442510|gb|AI822237.1|AI822237	GO:0006979 response to oxidative stress<br>GO:0006952 defense response<br>GO:0010224 response to UV-B<br>GO:0009611 response to wounding<br>GO:0006559 L-phenylalanine catabolic process<br>GO:0009699 phenylpropanoid biosynthetic process<br>GO:0009555 pollen development<br>GO:0046274 lignin catabolic process<br>GO:0009819 drought recovery<br>GO:0046244 salicylic acid catabolic process<br>GO:0080167 response to karrikin<br>GO:0006570 tyrosine metabolic process<br>GO:0009821 alkaloid biosynthetic process	GO:0005737 cytoplasm	GO:0045548 phenylalanine ammonia-lyase activity
21	gi|25802852|gb|CA758813.1|CA758813	GO:0009788 negative regulation of abscisic acid mediated signaling pathway<br>GO:0008360 regulation of cell shape<br>GO:0009414 response to water deprivation<br>GO:0048509 regulation of meristem development<br>GO:0018344 protein geranylgeranylation<br>GO:0018343 protein farnesylation<br>GO:0006694 steroid biosynthetic process<br>GO:0016114 terpenoid biosynthetic process	GO:0005965 protein farnesyltransferase complex<br>GO:0005953 CAAX-protein geranylgeranyltransferase complex	GO:0004662 CAAX-protein geranylgeranyltransferase activity<br>GO:0004660 protein farnesyltransferase activity<br>GO:0004311 farnesyltranstransferase activity<br>GO:0046982 protein heterodimerization activity
22	gi|209717502|gb|BW654405.1|BW654405	GO:0042218 1-aminocyclopropane-1-carboxylate biosynthetic process		GO:0030170 pyridoxal phosphate binding<br>GO:0008483 transaminase activity<br>GO:0016847 1-aminocyclopropane-1-carboxylate synthase activity
23	gi|209725557|gb|BW677859.1|BW677859	GO:0042218 1-aminocyclopropane-1-carboxylate biosynthetic process<br>GO:0006522 alanine metabolic process<br>GO:0006531 aspartate metabolic process<br>GO:0015976 carbon utilization		GO:0030170 pyridoxal phosphate binding<br>GO:0004021 L-alanine:2-oxoglutarate aminotransferase activity<br>GO:0016847 1-aminocyclopropane-1-carboxylate synthase activity
24	gi|56433440|gb|CX024350.1|CX024350	GO:0009098 leucine biosynthetic process<br>GO:0009651 response to salt stress<br>GO:0009097 isoleucine biosynthetic process<br>GO:0009099 valine biosynthetic process	GO:0009316 3-isopropylmalate dehydratase complex<br>GO:0009570 chloroplast stroma	GO:0003861 3-isopropylmalate dehydratase activity
25	gi|25799399|gb|CA755360.1|CA755360	GO:0009094 L-phenylalanine biosynthetic process		GO:0004664 prephenate dehydratase activity

		GO:0000162 tryptophan biosynthetic process<br>GO:0006571 tyrosine biosynthetic process		
26	gi|313127819|gb|HS106796.1|HS106796	GO:0006468 protein phosphorylation<br>GO:0007165 signal transduction<br>GO:0009069 serine family amino acid metabolic process		GO:0004697 protein kinase C activity<br>GO:0004713 protein tyrosine kinase activity<br>GO:0005524 ATP binding
27	gi|25799833|gb|CA755794.1|CA755794	GO:0009789 positive regulation of abscisic acid mediated signaling pathway<br>GO:0010111 glyoxysome organization<br>GO:0009611 response to wounding<br>GO:0009695 jasmonic acid biosynthetic process<br>GO:0006635 fatty acid beta-oxidation<br>GO:0006550 isoleucine catabolic process<br>GO:0006552 leucine catabolic process<br>GO:0006574 valine catabolic process<br>GO:0006699 bile acid biosynthetic process<br>GO:0018874 benzoate metabolic process<br>GO:0042967 acyl-carrier-protein biosynthetic process	GO:0005829 cytosol<br>GO:0005777 peroxisome<br>GO:0005739 mitochondrion<br>GO:0005730 nucleolus<br>GO:0005774 vacuolar membrane<br>GO:0009507 chloroplast	GO:0003988 acetyl-CoA C-acyltransferase activity
28	gi|21620941|gb|BQ618947.1|BQ618947	GO:0006550 isoleucine catabolic process<br>GO:0006552 leucine catabolic process<br>GO:0006574 valine catabolic process<br>GO:0006633 fatty acid biosynthetic process<br>GO:0006699 bile acid biosynthetic process<br>GO:0018874 benzoate metabolic process<br>GO:0042967 acyl-carrier-protein biosynthetic process	GO:0005777 peroxisome<br>GO:0005773 vacuole	GO:0003988 acetyl-CoA C-acyltransferase activity
29	gi|25798095|gb|CA754056.1|CA754056	GO:0042218 1-aminocyclopropane-1-carboxylate biosynthetic process<br>GO:0009853 photorespiration<br>GO:0006522 alanine metabolic process<br>GO:0006531 aspartate metabolic process<br>GO:0006544 glycine metabolic process<br>GO:0006563 L-serine metabolic process<br>GO:0006566 threonine metabolic process<br>GO:0015976 carbon utilization	GO:0005777 peroxisome<br>GO:0009570 chloroplast stroma<br>GO:0005829 cytosol	GO:0008453 alanine-glyoxylate transaminase activity<br>GO:0030170 pyridoxal phosphate binding<br>GO:0004021 L-alanine:2-oxoglutarate aminotransferase activity<br>GO:0047958 glycine:2-oxoglutarate aminotransferase activity<br>GO:0016847 1-aminocyclopropane-1-carboxylate synthase activity
30	gi|226763339|gb|GO519325.1|GO519325	GO:0009813 flavonoid biosynthetic process<br>GO:0009733 response to auxin stimulus<br>GO:0055114 oxidation-reduction process	GO:0005789 endoplasmic reticulum membrane<br>GO:0016021 integral to	GO:0016711 flavonoid 3'-monooxygenase activity<br>GO:0009055 electron carrier activity<br>GO:0020037 heme binding

		GO:0009411 response to UV<br>GO:0006118 electron transport	membrane	GO:0019825 oxygen binding
31	gi|49633098|gb|CO416850.1|CO416850	GO:0006412 translation<br>GO:0042254 ribosome biogenesis	GO:0005730 nucleolus<br>GO:0009506 plasmodesma<br>GO:0005886 plasma membrane<br>GO:0022625 cytosolic large ribosomal subunit<br>GO:0009507 chloroplast<br>GO:0005783 endoplasmic reticulum	GO:0003735 structural constituent of ribosome
32	gi|8334112|gb|BE039096.1|BE039096	GO:0009759 indole glucosinolate biosynthetic process<br>GO:0000162 tryptophan biosynthetic process<br>GO:0010114 response to red light<br>GO:0052544 defense response by callose deposition in cell wall<br>GO:0048830 adventitious root development<br>GO:0009411 response to UV<br>GO:0009684 indoleacetic acid biosynthetic process<br>GO:0009641 shade avoidance<br>GO:0055114 oxidation-reduction process<br>GO:0009682 induced systemic resistance<br>GO:0006118 electron transport	GO:0005783 endoplasmic reticulum<br>GO:0005886 plasma membrane<br>GO:0005739 mitochondrion	GO:0020037 heme binding<br>GO:0009055 electron carrier activity<br>GO:0033771 licodione synthase activity<br>GO:0019825 oxygen binding
33	gi|203711834|gb|GD250318.1|GD250318	GO:0006555 methionine metabolic process<br>GO:0055114 oxidation-reduction process<br>GO:0046653 tetrahydrofolate metabolic process		GO:0004489 methylenetetrahydrofolate reductase (NADPH) activity
34	gi|25802318|gb|CA758279.1|CA758279	GO:0006694 steroid biosynthetic process<br>GO:0016114 terpenoid biosynthetic process	GO:0016021 integral to membrane	GO:0004310 farnesyl-diphosphate farnesyltransferase activity
35	gi|219524684|gb|GH456542.1|GH456542	GO:0000162 tryptophan biosynthetic process<br>GO:0006571 tyrosine biosynthetic process<br>GO:0009094 L-phenylalanine biosynthetic process	GO:0009536 plastid	GO:0004834 tryptophan synthase activity
